# Case report: isolated prevotella intermedia causing intracranial infection detected using metagenomic next generation sequencing

**DOI:** 10.1186/s12883-023-03374-5

**Published:** 2023-10-23

**Authors:** Zhinan Ye, Jinfeng He, Hailong Ji, Hao Xu, Yaping Zhang, Kaiyu Zhou, Hongwei Liu

**Affiliations:** 1 Department of Neurology, Municipal Hospital Affiliated to the Medical School of Taizhou University, Taizhou, Zhejiang Province China; 2 Department of Neurosurgery, Municipal Hospital Affiliated to the Medical School of Taizhou University, No. 381 of East Zhongshan Road, Jiaojiang District, 318000 Taizhou, Zhejiang Province China; 3 Department of Neurology, Taiyuan Central Hospital, Shanxi Medical University, No.5, Three lanes East Road, Taiyuan, 030000 Shanxi Province China

**Keywords:** Prevotella intermedia, Metagenomic next generation sequencing, Intracranial infection, Cerebrospinal fluid, Magnetic resonance imaging

## Abstract

**Background:**

Isolated Prevotella intermedia, a rare gram-negative, rod-shaped, anaerobic bacterium, is rarely detected in clinical practice. It has been associated with infections of the oral cavity and female genital tract, but has never been detected in cerebrospinal fluid (CSF) of patients in China. Accurate detection of causative pathogens is still an arduous task owing to the difficult conditions of anaerobic bacterial culture. Isolated Prevotella intermedia can be detected by metagenomic next generation sequencing (mNGS) of the CSF. Correct diagnosis and antibiotic treatment can help patients avoid life-threatening events.

**Case presentation:**

Herein, we describe the case of a 64-year-old Chinese woman who presented with typical features of meningoencephalitis. Routine CSF culture failed to identify the causative pathogen. Isolated Prevotella intermedia was detected by mNGS, and the patient was treated with antibacterial agents including ceftriaxone, vancomycin, moxifloxacin, meropenem, metronidazole, and linezolid. The patient underwent surgical treatment for abscess of left frontal parietal lobe, which was observed on magnetic resonance imaging (MRI) and was suspected to be caused by Prevotella intermedia. It was further confirmed that it was a secondary infection from the oral cavity, and the possible etiology might have been dental surgery. Treatment was rendered to the patient based on metagenomic test result, and her condition improved after two months.

**Conclusions:**

This case highlights the role of mNGS in accurate diagnosis of patients with central nervous system infection. In particular, mNGS can be used to identify rare pathogens and confirm the diagnosis in patients with unknown etiology.

## Background

Prevotella intermedia are obligate anaerobic gram-negative bacilli, which form a part of the normal flora of the oral cavity, genito-urinary tracts and skin-soft tissue [1–3]. With the development of diagnostic technology in the recent years, isolation and timely identification of anaerobic bacteria is now possible. There have been several studies on identification and isolation of anaerobic bacteria related to oral cavity and female genital tract infections. However, only three adult cases of intracranial infection caused by isolated Prevotella intermedia (i.e., only one bacterium species) have been reported, one each from Japan, Korea and the United States of America (Table 1). Herein, we present a case from China to raise awareness regarding the severity of intracranial infection caused by Prevotella intermedia in clinical practice, to supplement the data of global epidemiology.

**Table 1:** Main features of reported cases of isolated prevotella intermedia-induced meningoencephalitis

Study/Year/ Country	Age/Sex	Bacteria	Location of MRI	Methods of identification	Treatment	Type of pathogeny	Outcome
Akimoto, et al. (2021) /Japan	51/F	Prevotella intermedia	Left frontal lobe	Surgical tissue culture	Piperacillin-tazobactam;metronidazole	N/A	Recovery
Park HR, et al. (2020) / Korea	47/M	Prevotella intermedia	Posterior fossa	16 S rRNA sequencing; CSF culture	Metronidazole	Chronic Sinusitis	Recovery
Brook I. (2010) / USA	16/M	Prevotella intermedia	Posterior fossa	CSF culture	Ceftriaxone;metronidazole	Rhinorrhea	Recovery

## Case presentation

**Fig. 1:**
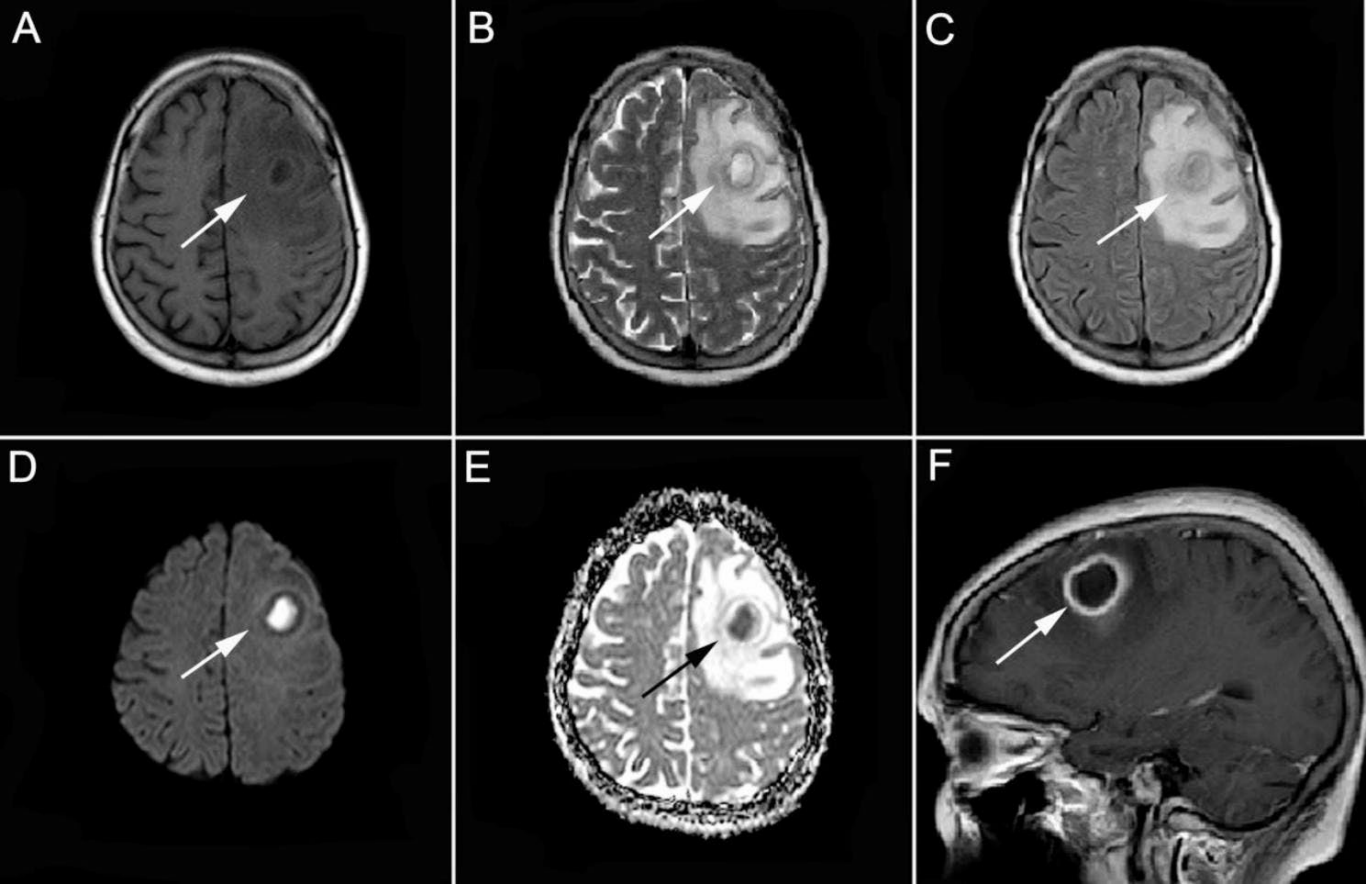
1.5-T MRI of brain abscesses before surgery. Typical imaging manifestations of brain abscesses were found in the left frontal and parietal region on coronal MRI slices with hypointensity on T1-weighted imaging (**A**) showing encephaledema. Hyperintensity on T2-weighted imaging (**B**), fluid-attenuation inversion recovery (FLAIR) imaging (**C**), and diffusion-weighted imaging (DWI) (**D**), as well as hypointensity on apparent diffusion coefficient (ADC) (**E**) imaging with encephaledema were also observed in brain abscesses—frontal and parietal lesions. Brain abscesses showed thin-rim enhancement with encephaledema on sagittal slices of MRI (**F**). White and black arrows indicate lesions

**Fig. 2:**
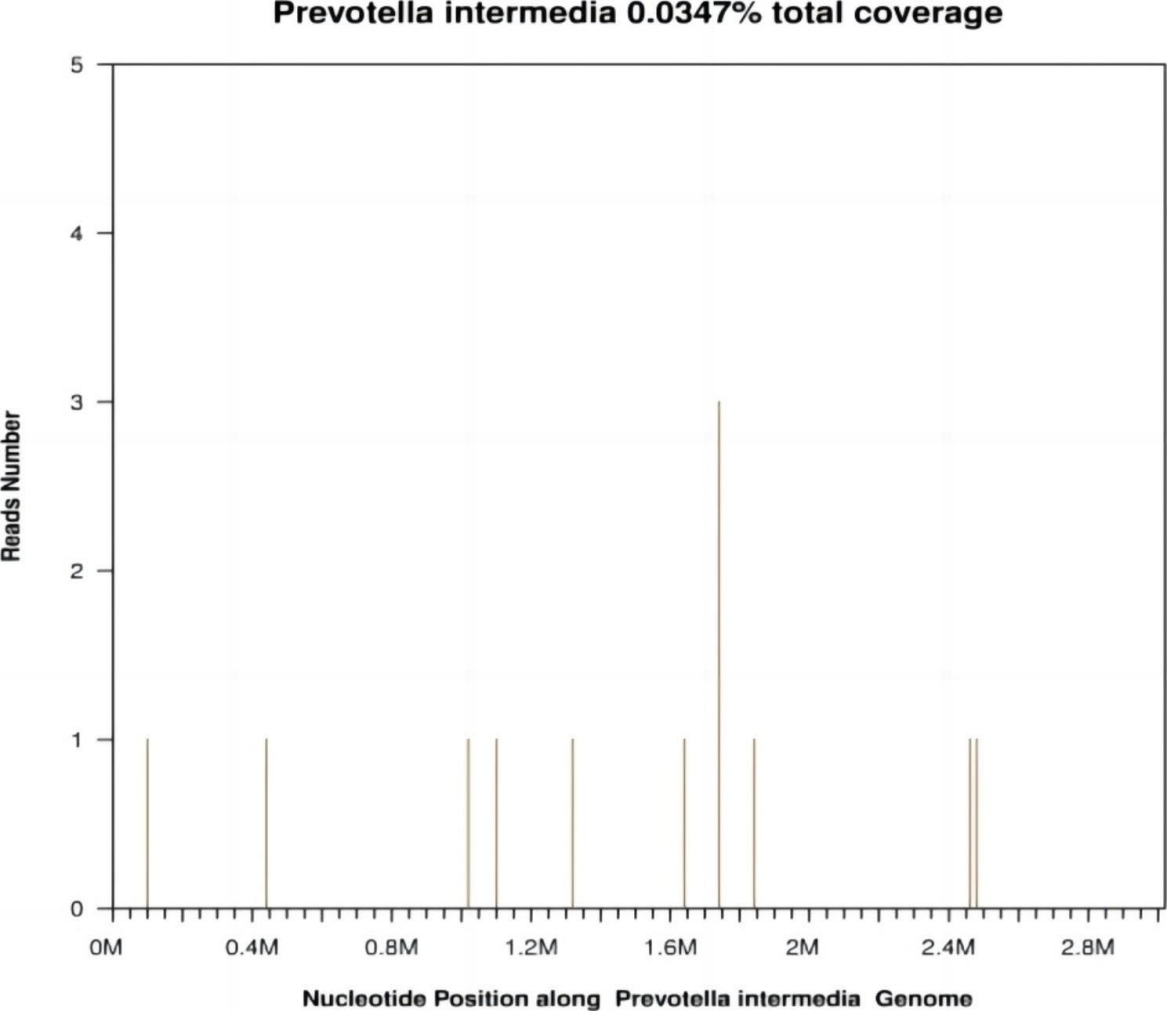
Sequence illustration of isolated prevotella intermedia causing intracranial infection detected by mNGS in CSF. Optimal thresholds for infectious Prevotella Intermedia identification by metagenomic next-generation sequencing (mNGS). The species identified by mNGS in CSF samples which had negative results by conventional culture and/or histopathology methods. A total of 12 reads were mapped to Prevotella intermedia in the reference database. The coverage of referenced Prevotella intermedia genome was 0.0347%. mNGS: metagenomic next generation sequencing

**Fig. 3:**
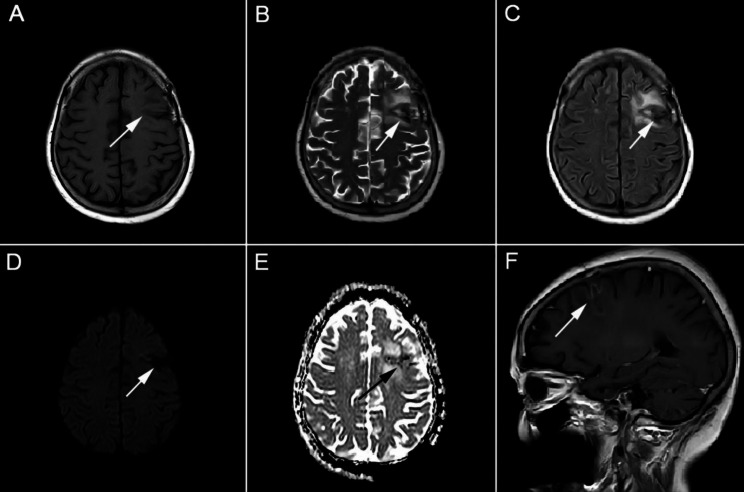
1.5-T MRI of brain at the 5 months follow up. Abnormal hypointensity on T1-weighted images (**A**), hyperintensity on T2-weighted images (**B**), and hypointensity on fluid-attenuation inversion recovery (FLAIR) images are observed in the brain lesions’ core (**C**). Additionally, hyperintensity around the brain lesions’ core on FLAIR imaging (**D**) suggested the softening of the lesions and secondary gliosis, as seen on coronal MRI slices. Hyperintensity on diffusion-weighted imaging (DWI) in the brain lesions disappeared (**D**) and hyperintensity on apparent diffusion coefficient (ADC) imaging increased (**E**), as seen on coronal MRI slices. Brain abscesses showed slight enhancement, as seen on the MRI sagittal image (**F**). White and black arrows indicate lesions

A 64-year-old Chinese woman reported to the emergency department with a history of headache for 3 days. She was previously healthy and had no drug history. For the treatment of periodontitis, caries, and pulpal necrosis, the patient received dental fillings and antibiotics approximately 2 weeks before the onset of the disease. Unfortunately, the patient did not comply with the doctor’s recommended treatment plan and only took oral metronidazole tablets for 5 days (600 mg q8h) instead of the full 2-week course. After admission, she complained of worsening and pulsating headache in the left orbit and frontotemporal region. On examination, she had a temperature of 38.8℃ and showed stiffness of the neck. Laboratory examination revealed white blood cell count of 9,500/mL with 75.3% neutrophils and 16.7% lymphocytes; hemoglobin value of 11.3 g/dL, and a platelet count of 4,35,000/mL. The C-reactive protein level was 57.35 mg/L. Cranial computed tomography and 1.5-Tesla (T) magnetic resonance imaging (MRI) showed no abnormalities. The patient was administered intravenous levofloxacin (500 mg qd) for her headache, which did not completely resolve the symptom. Her headache gradually worsened, she became less responsive, and showed signs of mental weakness with reduced appetite. Due to the severity of her symptoms, the patient was rushed to Taizhou Central Hospital for further treatment. There, a lumbar puncture (LP) was performed, which demonstrated colorless and clear fluid with protein level of 84 mg/dL and glucose level of 82 mg/dL. White blood cell (WBC) count in the cerebrospinal fluid (CSF) was 800/mm^3^ (reference range 0 ~ 8 × 10^3^/mm3), with 100% peripheral blood mononuclear cells. Based on the CSF reports and clinical characteristics, an initial diagnosis of herpes simplex encephalitis was made, and the patient was treated with 10 days of intravenous acyclovir (500 mg q8h). However, on the 10th day of hospitalization, her headache, nausea and vomiting gradually aggravated, and she presented with horizontal diplopia which implied left abducens nerve palsy. Additionally, the patient had persistent high fever and an episode of non-projectile vomiting. A second LP was performed on the 10th day of hospitalization, which revealed WBC count of 400/mm^3^ in the CSF. The number had decreased slightly compared to the previous LP. Gram staining for bacteria was negative. Samples of blood, urine, and CSF were sent for culture. Considering the poor effect of antiviral treatment, differential diagnoses of atypical or rare bacterial meningoencephalitis (BME) were considered. The patient received the following empirical antibiotics with intravenous ceftriaxone (3000 mg qd), metronidazole (500 mg q12h), and vancomycin (500 mg q12h) for BME, and was admitted to the intensive care unit for further management. Two weeks later, her condition improved. However, her condition worsened again after 21 days of hospitalization. Physical examination revealed unequal pupil sizes, with the left pupil not being round and showing weakened reaction to light. The second 1.5-T MRI of brain showed abscess in the left frontal and parietal lobe, and left frontal craniotomy was performed for abscess removal (Fig. 1). When bur hole drainage was performed for the brain abscess, approximately 19 mL of pus was aspirated. Postoperative brain histopathology revealed left frontal subpial inflammatory lesions. Serum (1,3)-β-D glucan (G test) and galactomannan (GM test) were negative. CSF ink staining and acid-fast bacilli smear were also negative. Serological tests for Borrelia burgdorferi, Treponema and Pallidum, iso-electric focusing, and viral PCR (Herpes Simplex, Varicella-Zoster, Epstein-Bar, Cytomegalovirus) were reassuring. The third CSF culture also did not reveal any bacterial growth, and no pathogenic microorganisms were found in the tissues from intracranial abscesses and and pus. Therefore, metagenomic next generation sequencing (mNGS) of the CSF was performed using the processing platform of the experimental data in Shanghai MedcareDx Biotechnology Co., Ltd., which revealed isolated Prevotella intermedia (Fig. 2). Based on clinical signs and symptoms, combined with CSF mNGS results, a diagnosis of anaerobic BME was made. A modified antibiotic treatment regimen (vancomycin [500 mgq12h iv], meropenem [1000 mg q8h iv], and moxifloxacin [400 mg qd iv]) was administered for approximately two months. In addition, she was transferred to another hospital where she continued to receive infusion treatment with moxifloxacin and linezolid. During the next 5 months of follow-up, dynamic re-examination using 1.5-T cranial MRI showed that the absorption of intracranial abscess was better than before (Fig. 3). The patient made a complete recovery.

## Discussion and conclusions

To the best of our knowledge, Prevotella intermediate and Streptococcus constellations cause mixed intracranial infections due to the difficulty of anaerobic bacterial isolation and need for specific methods, but which of these plays an important role in anaerobic infections remains unclear [4]. Our study addresses this puzzling question by focusing on isolated Prevotella intermedia to identify the cause of intracranial infection. This is the first reported case of intracranial infection caused by isolated Prevotella intermedia from China. This case report highlights the role of mNGS in atypical meningoencephalitis cases. Anaerobic pathogens in adult BME are very rare, and therefore, often misdiagnosed and treated incorrectly [5]. The use of mNGS would aid in accurate identification, and apt treatment, so that life-threatening conditions can be avoided.

Prevotella, previously classified in the genus Bacteroides, is a genus of an obligate anaerobic gram-negative rod-shape bacterium. Many Prevotella taxa from oral cavity are potential/ pportunistic pathogens. Prevotella intermedia causes oral diseases, such as periodontal disease, periapical periodontitis, dental caries, and periodontal abscesses [6]. Prevotella intermedia colonizes the respiratory tract and to be associated with cystic fibrosis and chronic bronchitis [7, 8]. It has also been observed in the oral cavities of women with reduced salivary secretions and was found to be associated with Sjögrens syndrome [9]. Additionally, Prevotella intermedia has been found in the oral cavities of patients with squamous cell carcinoma of the head and neck and was proven to be enriched in tumor tissues [10, 11]. Prevotella intermedia are also reportedly found in various types of abscesses at different body sites, including the brain, neck, lungs, breast, pleura, subdural region, and soft tissues [6, 12]. Limited information is available on the involvement of Prevotella organisms under vascular conditions in the intracranial region. Regarding ischemic stroke, Prevotella intermedia has been proven to be enriched in patients with poor treatment outcome [13]. It has also been cultured in the cerebrospinal fluid of a patient with intracranial mycotic aneurysm [14]. Prevotella intermedia meningitis has been associated with cerebrospinal fluid leakage in an adolescent, with the suggestion that its route of extension could be through retrograde influx from the oropharynx into the cerebrospinal space [15].

The patient underwent dental surgery approximately two weeks before the onset of the disease, which are the typical predisposing conditions for anaerobic bacterial meningoencephalitis. Isolated Prevotella intermedia can cause various oral disorders like periodontal diseases, periapical periodontitis and noma [16, 17]. Bacterial infections theoretically spread from oral cavity to central nervous system (CNS) via one of four routes: (i) inoculation via contiguous extension or by introduction of foreign objects; (ii) systemic hematogenous bacteremia; (iii) facial and pterygoid vein systems; and (iv) lymphatic drainage. Habibian et al. reported that prevalence of intra oral infection and the concordance of cavernous sinus lesions and it flows into the cranial fornix through the facial and pterygoid systems [18]. In this case, presence of typical susceptibility conditions for anaerobic BME lead the clinicians to consider the possibility of BME associated with anaerobic bacterial infection of the oral cavity.

Culturing anaerobic bacteria in clinical microbiology laboratories is often limited due to the following reasons: (i) strict culture environment requirements; (ii) culture is time-consuming and easily lost during the passaging process; (iii) identification of rare species is time-consuming and unreliable; (iv) difficulty associated with assessment of correlation between anaerobic bacteria and other flora [19]. Continuous development and progress in clinical microbiological diagnostic technology has made the culture and identification of anaerobic bacteria more convenient and rapid. The current identification methods of anaerobic bacteria include: automated bacterial identification instrument, 16 S ribosome ribonucleic acid (16 S rRNA), matrix-assisted laser desorption/ionization time of flight mass spectrometry (MALDI-TOF MS) and mNGS. Among these, the first two identification methods rely on conventional bacterial culture, while 16 S rRNA and mNGS can directly identify the bacteria. With the emergence of mNGS technology, all nucleic acid sequences in the sample can be amplified using random primers. Theoretically, all potential pathogens can be detected without bias, especially rare, new or atypical pathogens of complex infectious diseases. mNGS has the advantages of high sensitivity, strong accuracy and short detection time.

Li et al. tested 29 microbiological samples of patients clinically diagnosed with anaerobic infection and reported that the positive rate of mNGS (82.76%) was higher compared to bacterial culture (20.76%) [20]. Moreover, the average detection time of mNGS is shorter than that of bacterial culture. However, this technology also has certain limitations, including lack of culture findings and inability to perform drug susceptibility tests on the detected pathogens. Moreover, it is difficult to distinguish whether the detected pathogens are a result of infection, colonization, or contamination [21]. In a previous study, only one of eight cases showed presence of gram-negative bacilli and gram-positive cocci in CSF smear, while the remaining cases of CSF culture and smear were negative. However, mNGS could detect the pathogenic bacteria in CSF of all eight patients [22]. In the present case, when the CSF, blood, urine, and biopsy brain tissue cultures were all negative, mNGS of CSF confirmed that the pathogen was isolated Prevotella intermedia. In clinical practice, reasons for failure of detection include: (i) Gram staining is not sensitive enough, and requires a large amount of bacteria to be visible under the microscope; (ii) standard culture methods may cause false negatives due to various reasons, such as specimen collection after antibiotic administration, unsuitable culture environment for microbial growth.

Our report shows that Prevotella intermedia may not only mimic the clinical pattern, but also the CSF characteristics of herpes simplex virus. If the patient’s response to antiviral therapy is poor, it can be considered that patients with such clinical presentation may already have anaerobic infections. In addition to herpes simplex encephalitis, similar imaging and clinical features have been reported in cases of Nocardia, actinomyces, tuberculosis, fungal infections, and tumors in previous studies. Therefore, for cases similar to the present case, the following differential diagnoses can be considered. (i) Actinomycosis: Actinomycosis of the central nervous system (CNS) should be considered in patients with a history of actinomycosis in another part of the body [23]. Symptoms are frequently of long duration, and fever is commonly absent [23]. Patients usually present with minimal, nonspecific, constitutional symptoms and an intracranial mass mimicking “brain space-occupying lesions,” such as abscesses or tumors [24]. Because incubation for at least 10 days in strictly anaerobic culture conditions is required for the isolation of Actinomyces species, a confirmative diagnosis of actinomycosis is difficult. Therefore, the 16 S rRNA gene sequencing method represents a reliable alternative for identification [24]. (ii) Nocardiosis: Brain abscesses due to Nocardia are rare and are usually found in immunocompromised patients. Unlike other bacterial abscesses, clinical manifestations of Nocardia brain abscesses have been reported and were usually insidious, without fever or signs of septicemia. The most frequent symptoms at presentation were headache, altered mental status, and focal neurological deficits [25]. Diagnosis of brain abscess is based mainly on bacteriological cultures from pus collected at the site of infection, and brain imaging. However, Nocardia spp. have relatively slow growth, and they can be difficult to culture in the laboratory, making the 16 S rRNA gene sequencing method a reliable alternative diagnostic method [26]. (iii) Mycosis: An increase in fungal brain abscesses could be attributed to the rise in immunosuppression, owing to the use of steroids and immunosuppressants for various disorders, HIV infection, and solid organ transplantation. CNS involvement typically starts in the lung or sinuses due to spore inhalation [27]. Patients typically present with neurological symptoms such as mental status changes, headaches, seizure, focal neurologic deficits, and encephalopathy [28]. Fungal abscesses have certain imaging features, such as number (often multiple) and location (they tend to involve the deep gray nuclei unlike pyogenic abscesses which more often spare the basal ganglia) [29]. Definitive diagnosis is by biopsy or culture and is usually difficult and delayed. (iv) Tuberculosis: Tuberculosis is responsible for almost all cases of intracranial tubercular infection.Patients may present acutely with fever, headache, focal neurological deficits corresponding to the site of the tuberculous brain abscess, with surrounding edema, or with signs and symptoms of raised intracranial pressure, which deteriorate more rapidly in comparison to patients with tuberculomas [30]. The radiological presentation usually is a uni- or multi-loculated lesion with a thin and uniform capsule, although it may be indistinguishable from pyogenic abscesses [31]. Diffusion and proton MR spectroscopy have been found to be effective in differentiating pyogenic from tuberculous brain abscesses which display a characteristic lipid peak [32]. The positive CSF culture represents strong evidence for the diagnosis of tuberculous brain abscesses.

Diagnosis is often difficult and imprecise and requires a combination of clinical features, radiological studies, and laboratory investigations. Using clinical assessment combined with brain imaging and CSF investigations, clinicians should be able to make a prompt diagnosis of possible or probable with a high index of clinical suspicion to avoid delays in starting appropriate treatment. Because actinomycosis, tuberculosis, and nocardiosis mimic malignancy and progress continuously, their diagnosis is often challenging and delayed. A correct preoperative diagnosis is essential for the treatment and prognosis of CNS tumors and brain abscesses, but the differentiation between them remains challenging. Thus, an accurate diagnosis can be achieved after evaluating a combination of clinical, radiological, and pathological criteria.

Cephalosporins (95%), nitroimidazole (72%), carbapenem (31%) and glycopeptide (28%) antibiotics are the most commonly used antibacterial agents for the treatment of anaerobic infections of the CNS [33]. Castillo et al. previously reported on the antibiotic susceptibility and presence of resistance genes in clinical oral isolates of Prevotella intermedia. They found the frequency of resistance gene in Prevotella intermedia (species) to be 30.2% [34]. Furthermore, Prevotella intermedia was resistant to metronidazole at a frequency of 30%; however, no isolates of Prevotella intermedia were resistant to amoxicillin/clavulanic acid, tetracycline, or clindamycin [34]. Notably, Prevotella intermedia ZT belongs to a genus marked with highly dynamic genomes. The specific genes of Prevotella intermedia contribute to its adaptation and pathogenic, and indicate that adhesion, competing with surrounding microbes, and horizontal gene transfer are the main drivers of the evolution of Prevotella intermedia [35]. However, It is difficult to demonstrate the relationship between clinical treatment failure and antibiotic resistance in anaerobic infections. Several factors may play important roles in the treatment of anaerobic infections, including synergy between bacteria, time of initiation of treatment, concentration of antibiotics at the site of infection, and underlying co-morbidities of the patient [36]. In addition, due to the presence of a unique blood-brain barrier, the concentration of antibiotics in the CNS is relatively low. Therefore, it is necessary to search for antibiotics with good blood-brain barrier penetration and bactericidal power for the treatment of anaerobic infections of the CNS.

Chinese experts recommend that patients with mild illness should first undergo traditional microbiological examination of CSF, and mNGS can be carried out when the etiology is not clear. For patients with encephalitis, meningitis and/or brain abscess of unknown etiology, poor treatment effect, severe disease and immune deficiency, it is recommended that mNGS detection be carried out immediately [37]. Clinicians should choose an appropriate time to test microbial samples, based on their clinical experience. If the samples are tested by mNGS after rendering treatment, false negative results may be obtained. In summary, when a patient presents with headache, fever and has potential risk factors, anaerobic infection of the CNS should be considered. Routine and anaerobic culture of CSF samples should be carried out, and if necessary, mNGS should be performed for definitive diagnosis. Prompt antimicrobial therapy is critical for patient recovery. This case report would increase the global awareness of severity of isolated Prevotella intermedia-induced intracranial infections.

## Data Availability

The data that support the findings of this study are available from the corresponding author.

## Abbreviations

CSF: Cerebrospinal fluid
mNGS: Metagenomic next generation sequencing
MRI: Magnetic resonance imaging
LP: lumbar puncture
WBC: White blood cell
BME: Bacterial meningoencephalitis
16S rRNA: 16 S ribosome ribonucleic acid
MALDI-TOF MS: matrix-assisted laser desorption/ionization time of flight mass spectrometry
CNS: central nervous system
